# Data Sharing Goals for Nonprofit Funders of Clinical Trials

**DOI:** 10.2196/23011

**Published:** 2021-03-29

**Authors:** Timothy Coetzee, Mad Price Ball, Marc Boutin, Abby Bronson, David T Dexter, Rebecca A English, Patricia Furlong, Andrew D Goodman, Cynthia Grossman, Adrian F Hernandez, Jennifer E Hinners, Lynn Hudson, Annie Kennedy, Mary Jane Marchisotto, Lynn Matrisian, Elizabeth Myers, W Benjamin Nowell, Brian A Nosek, Todd Sherer, Carolyn Shore, Ida Sim, Luba Smolensky, Christopher Williams, Julie Wood, Sharon F Terry

**Affiliations:** 1 National Multiple Sclerosis Society Cherry Hill, NJ United States; 2 Open Humans Foundation San Diego, CA United States; 3 Novartis Basel Switzerland; 4 Edgewise Therapeutics Boulder, CO United States; 5 Parkinson’s UK London United Kingdom; 6 National Academies of Sciences, Engineering, and Medicine Washington, DC United States; 7 Parent Project Muscular Dystrophy Bethesda, MD United States; 8 Department of Neurology University of Rochester Medical Center Rochester, NY United States; 9 Biogen Cambridge, MA United States; 10 Duke University School of Medicine Durham, NC United States; 11 Bison Ink Washington, DC United States; 12 Critical Path Institute Tucson, AZ United States; 13 MJM Advisory, LLC New York, NY United States; 14 Pancreatic Cancer Action Network Washington, DC United States; 15 Doris Duke Charitable Foundation New York, NY United States; 16 Global Healthy Living Foundation Upper Nyack, NY United States; 17 Center for Open Science Charlottesville, VA United States; 18 The Michael J Fox Foundation for Parkinson’s Research New York, NY United States; 19 Department of Medicine University of California San Francisco San Francisco, CA United States; 20 Multiple Myeloma Research Foundation Norwalk, CT United States; 21 Vivli Columbia, MO United States; 22 Genetic Alliance Washington, DC United States

**Keywords:** clinical trial, biomedical research, data sharing, patients

## Abstract

Sharing clinical trial data can provide value to research participants and communities by accelerating the development of new knowledge and therapies as investigators merge data sets to conduct new analyses, reproduce published findings to raise standards for original research, and learn from the work of others to generate new research questions. Nonprofit funders, including disease advocacy and patient-focused organizations, play a pivotal role in the promotion and implementation of data sharing policies. Funders are uniquely positioned to promote and support a culture of data sharing by serving as trusted liaisons between potential research participants and investigators who wish to access these participants’ networks for clinical trial recruitment. In short, nonprofit funders can drive policies and influence research culture. The purpose of this paper is to detail a set of aspirational goals and forward thinking, collaborative data sharing solutions for nonprofit funders to fold into existing funding policies. The goals of this paper convey the complexity of the opportunities and challenges facing nonprofit funders and the appropriate prioritization of data sharing within their organizations and may serve as a starting point for a data sharing *toolkit* for nonprofit funders of clinical trials to provide the clarity of mission and mechanisms to enforce the data sharing practices their communities already expect are happening.

## Introduction

### Clinical Trial Data Sharing and the Role of Nonprofit Funders

Sharing clinical trial data can provide value to research participants and communities by accelerating the development of new knowledge and therapies as investigators merge data sets to conduct new analyses (eg, meta-analyses, statistical modeling), reproduce published findings to raise standards for original research, and learn from the work of others to generate new research questions.

Nonprofit funders, including disease advocacy and patient-focused organizations, play a pivotal role in the promotion and implementation of data sharing policies. Funders are uniquely positioned to promote and support a culture of data sharing by serving as trusted liaisons between potential research participants and investigators who wish to access these participants’ networks for clinical trial recruitment. In short, nonprofit funders can both drive policies and influence research culture.

In the US Institute of Medicine (IOM) 2015 report, *Sharing Clinical Trial Data: Maximizing Benefits, Minimizing Risk*, nonprofit funders were specifically identified as key stakeholders for advancing data sharing, while the important role of clinical trial participants, themselves, was additionally recognized [1]. As stated in the report, “the movement towards greater transparency is being further accelerated by trial participants...and a larger cultural shift already underway...in which the results of research are deemed a public good that can benefit society only when shared in a timely and responsible manner.”

Despite arguments to the contrary, in many cases, clinical trial participants are indeed willing to share their data for a wide range of uses, assuming that adequate security safeguards are in place [2]. Patients may assume that data sharing is already taking place and could become frustrated when they learn that many nonprofit organizations and academic researchers are not actively implementing data sharing policies. Patient communities rightfully expect that the nonprofit funders who encourage them to enroll in studies would also take action to ensure that data resulting from these trials are shared.

### The Current State of Clinical Trial Data Sharing

Since the release of the 2015 IOM report, several efforts in the public sector have signaled that data sharing is increasingly regarded as a scientific responsibility rather than an optional activity [3-5]. For example, the bipartisan 21st Century Cures Act signed into law in December 2016 contains a number of provisions focused on advancing the responsible sharing of clinical trial data from government-funded research and improving public interfaces such as ClinicalTrials.gov [6]. ClinicalTrials.gov has since implemented changes to facilitate centralized access to and the discoverability of individual participant data (IPD) sharing plans and whether and where IPD and supporting information (eg, clinical study reports) are available after study completion [7]. In 2016, the FAIR Data Principles were published, providing guidelines for making scientific data FAIR—findable, accessible, interoperable, and reusable [8]. The GO FAIR Initiative was also established to help implement these guidelines, focusing on early developments in the European Open Science Cloud [9]. The International Committee of Medical Journal Editors also released policies requiring increased transparency and prioritization of data sharing among the manuscripts submitted to their journals. The Wellcome Trust released a policy on managing and sharing research outputs that include data, software, and materials [10-12].

More recently, for public comment, the National Institutes of Health (NIH) released an updated Draft NIH Policy for Data Management and Sharing, which would require NIH-funded grantees to submit a data management and sharing plan on how researchers intend to preserve and share their data [13-15]. In addition, clinical trial data sharing platforms such as Vivli, the Yale University Open Data Access Project, Supporting Open Access for Researchers, and ClinicalStudyDataRequest.com have enabled researchers and sponsors seeking share and access data from clinical trials [16-18]. Clinical trial data sharing workshops, such as the National Academies’ recent 2019 workshop, *Sharing Clinical Trial Data: Challenges and a Way Forward*—have also helped advance the dialog by highlighting challenges, successes, and next steps in data sharing endeavors [19].

In 2015, following the publication of the IOM consensus study, *Sharing Clinical Trial Data: Maximizing Benefits, Minimizing Risk*, Tudur Smith et al [20] published a set of *good practice principles* for data sharing, which emphasized controlled and secure access, participant consent and confidentiality, and a multistakeholder approach for supporting the required resources [1]. Additional publications since this time have included the development of data sharing principles for specific diseases such as the Alzheimer disease, critiques of data access review policies, and proposed strategies for implementing protected cloud-based methods of clinical trial data sharing [21-23]. In a more comprehensive critique on data sharing and the reuse of individual participant-level data from clinical trials, Ohmann et al [24] published a number of principles and recommendations that resulted from a multistakeholder consensus process. Although these principles and recommendations are not necessarily targeted for nonprofit funders, there may be potential applications for funders, as is reflected in this data sharing paper.

Other recent publications have more specifically assessed and advised the practices of funders in data sharing. In one retrospective review published by Whitlock et al [25], clinical trial transparency policies were evaluated among the top 10 noncommercial US health researcher funders. The overall proportion of US funders with policies and practices to support transparency was found to be similar to that of larger international noncommercial funders. Terry et al [26] have additionally provided the following few key recommendations specific to funders to support the sharing of data: (1) funders should engage early with researchers to understand their concerns and more explicitly define the benefits for all stakeholders, (2) there should be a direct benefit to sharing data relevant to those people who collect and curate the data, and (3) a checklist of issues to be addressed should be developed for designing or revising data sharing resources.

### Challenges and Goals for Nonprofit Funders

Although data sharing has gained momentum, major challenges remain, including transparency of data access procedures, reuse of consent provisions (ie, policies that accommodate participant consent regarding the volume, nature, and timing for secondary use of clinical trial data for future scientific research), governance of data sharing policies, availability of affordable technical infrastructure, alignment on data standards, and a highly fragmented landscape of data repositories [27,28]. In particular, for nonprofit funders, data sharing can be a resource-intensive activity, requiring investment trade-offs between data sharing and other research priorities. In terms of bargaining power, nonprofit funders often have limited capability to enforce policies and contracts with individual investigators, despite having provided financial support for research projects.

The purpose of this paper is to detail a set of aspirational goals and forward thinking, collaborative solutions to data sharing for nonprofit funders to fold into existing funding policies. This paper was developed by the Sharing Clinical Trial Data Action Collaborative, an ad hoc activity associated with several forums at the National Academies of Sciences, Engineering, and Medicine: the Forum on Drug Discovery, Development, and Translation; the Roundtable on Genomics and Precision Health; the Forum on Neuroscience and Nervous System Disorders; and the National Cancer Policy Forum. The paper does not necessarily represent the views of any one organization, the Forums or Roundtable, or the National Academies and has not been subjected to the review procedures of, nor is it a consensus study report or product of, the National Academies.

Throughout this paper, *nonprofit funders* refers to both traditional nonprofit funders (such as foundations and philanthropic organizations) and the full spectrum of nonprofit patient-focused and disease-advocacy organizations that fund or otherwise support clinical trials and *grantees* refers to those individuals, groups, institutions, and organizations who receive funding or other support for clinical trials. Clinical trial data may take many forms, including IPD (ie, raw data and the analyzable data set); metadata, or the information required to contextualize and understand a given data element; and summary-level data. Data may be identifiable or deidentified. *Data sharing* within this paper refers to the process of making clinical trial data—particularly IPD—available to secondary users, and *shared data* refers to any data accessed as a result of data sharing policies and processes. Recognizing that a range of contracting arrangements are possible, *grantees* refers to those receiving funds from nonprofit organizations and could be either (1) the research institution as a whole or (2) an individual researcher. Although this paper aims at data from prospective, interventional clinical trials, many of the goals and illustrative examples encompass clinical research more broadly.

## Sharing Clinical Trial Data Action Collaborative

To discuss and advise on the development of data sharing goals for nonprofit funders of clinical trials, an ad hoc group of individuals with relevant expertise representing nonprofit organizations and other stakeholders, including patient and disease advocacy representatives, researchers, regulators, and drug developers, was convened in July 2016 (n=9) and November 2017 (n=39) in Washington, DC, associated with the Sharing Clinical Trial Data Action Collaborative of the National Academies of Sciences, Engineering, and Medicine. Invited participants were identified by project coleaders, Sharon Terry (Genetic Alliance) and Timothy Coetzee (National Multiple Sclerosis Society), and with the research assistance of National Academies staff, to represent a subset of nonprofit funders of clinical trials interested in exploring and implementing data sharing policies. Over the course of planning for the 2016 and 2017 discussion meetings, 70 individuals were invited and 48 accepted and participated in either or both the 2016 and 2017 meetings.

The 2016 meeting focused on gathering feedback on a draft set of data sharing principles for nonprofit funders drafted by Sharon Terry, Tim Coetzee, and National Academies staff working with Action Collaborative and drawn largely from themes in the 2015 IOM report, *Sharing Clinical Trial Data: Maximizing Benefits, Minimizing Risk* [1]. On the basis of the 2016 discussion meeting, the principles were refined, including changing *principles* to *goals*, and presented to the larger group of stakeholders at the 2017 meeting. The 2017 discussion led to additional rounds of editing and refining by email among the meeting participants to reach the paper presented here. The project coleaders made clear to meeting participants that all views—supportive or not—of data sharing were welcome throughout the process.

The National Academies provided a neutral venue for nonprofit funders to have a candid conversation about the opportunities and challenges of taking up formal data sharing policies within their organizations. Participants in this activity had an opportunity to share perspectives, sharpen questions, spark new ideas, and explore possible solutions. The participants did not cover a full representation of the nonprofit community. The goals included in this paper are not binding agreements but rather indications of support for advancing clinical trial data sharing among the nonprofit community.

### Data Sharing Goals for Nonprofit Funders of Clinical Trials

#### Goal #1: Encourage Co-Development of Data Sharing Policies With Patient and Lay Communities

Patient communities and the lay public should have a voice in the development of data sharing policies regarding what and how data will be shared with others, including, but not limited to, IPD, participant-generated data (eg, patient-reported outcomes, patient data acquired from wearables), lay summaries, and resulting publications [29]. Members of the lay public should be codevelopers of all aspects of clinical research, including, but not limited to, clinical trial data sharing programs. The input and participation of patient communities and the lay public should infuse the entire process and be clearly stated in the informed consent process; it cannot be reverse engineered.

Embedded in this goal is the collective duty of nonprofit funders to prioritize educating patient communities about data sharing and build an informed public trust around the value of clinical trials and data sharing.

#### Goal #2: Incorporate Data Sharing Concepts and Policies as Early as Possible in the Clinical Trial Process

Nonprofit funders should strive to prioritize data sharing in the earliest conceptions of a clinical trial. The ultimate utility of shared clinical trial data often hinges on the degree to which sharing was planned for from the beginning—a principle also endorsed by the multistakeholder task force on data sharing, as described by Ohmann et al [24]. When sharing is planned well in advance, researchers can ensure that data are collected and prepared in a way that enables effective sharing.

Nonprofit funders can incorporate data sharing principles into grant application policies. For example, grant provisions could require that the proposed clinical trials include a plan for how and when data from the trial will be shared and that clinical trials be registered on a public data sharing platform (eg, ClinicalTrials.gov). In addition, nonprofit funders may set expectations for awardees to deposit clinical trial data in public databases and publish results of the trial regardless of the outcome.

#### Goal #3: Develop or Adopt Transparent and the FAIR Approval Processes for Data Access

Nonprofit funders should work with grantees and patient communities to ensure that data access policies (1) facilitate the appropriate use of shared data, (2) enable research participants’ data access preferences, (3) protect participant privacy, and (4) mitigate risks to the scientific integrity of investigators and sponsors that share data (ie, reducing the likelihood of misuse or misanalysis of shared data)—all without unduly restricting access to shared data. This goal echoes broader data sharing goals of making data access processes more explicit and transparent, and funder actions made to achieve this goal would additionally demonstrate support and alignment with FAIR principles [24,25].

Data access policies may vary based on the type of data being collected in a clinical trial and the preferences of the community. For instance, IPD with imaging and genetic information may warrant a third-party intermediary to review requests, as opposed to open access for anyone. It is difficult to make one-size-fits-all decisions on who should be authorized to have access to data, but variations are possible. For instance, platforms and technology exist to enable individuals to decide who should access their data.

Nonprofit funders could encourage or require grantees to provide the following information and respect the following policies as objective signals of the FAIR use and intent:

1. Establishing a plan or proposal that states the purpose of the data request (eg, to support hypothesis generation or protocol development)
2. Providing evidence via a standard biographical sketch of the qualifications of the requestor
3. Using data use agreements that may help ensure that data requesters follow the plan stated in the original request and do not attempt to use data in harmful or malicious ways, such as reidentifying participant data or using data for commercial or litigation purposes
4. Sharing clinical trial data, particularly of sensitive nature, in a way that it can be housed, accessed, and analyzed behind a firewall or other secure mechanism
5. Third-party review teams can vet data requests to provide an independent, transparent, accountable, and efficient data access review process (Textbox 1).

**Considerations for third-party teams reviewing data requests.**

**Third-party review teams could consider the following criteria when reviewing requests for data:**

1. Can the data requested support the stated purpose of the requestor?
2. Does the request have a public health or health goal and address real patient needs?
3. What technology infrastructure is used to provide data access, and who is the data steward?
4. What data standards are used to share the final data set?
5. What data versioning processes does your data steward recommend?
6. What data variables and data types are shared and over what length of time?
  What security measures are required for your shared data?
  What is detailed in the data documentation (ie, data dictionary, schemas, example analyses, and use)?

#### Goal #4: Promote the Development of a Sustainable and Feasible Data Sharing Infrastructure

Requiring that data be shared but not providing a place to host shared data is an impractical mandate, and the recognition of the general need to properly structure and build a suitable and sustainable infrastructure for data sharing has remained an important recognition [24]. Consequently, an increasing number of platforms for data storage, curation, sharing, and archiving exist or are in development. For example, the Genetic Alliance established Promise for Engaging Everyone Responsibly (PEER) to help advocacy organizations, nonprofits, clinics, and sponsors establish data collection registries [30]. PEER enables participants to securely upload and store data (eg, electronic health records, health surveys, genomic and genetic information) and decide what data they will share. Companies and researchers can then access data to carry out study analysis with the appropriate embargos and mechanisms to release data back to the individuals and communities.

Many nonprofit funders acknowledge that developing and maintaining the technology to support a data repository is beyond their skill set. Thus, nonprofit funders will increasingly be looking to external platforms—and partnerships with technology companies—for hosting shared data. Nonprofit funders should work together to collectively form partnerships and provide support (eg, funding, guidelines, and requirements) for data sharing platforms in accordance with the needs and goals of their communities. Although each community will still have unique needs and expectations, there are significant similarities among the desired specifications of a data sharing infrastructure and much to be gained from ensuring that data are not unnecessarily siloed.

Within their organizations, nonprofit funders should develop and implement data sharing guidelines and/or policies that detail requirements for storage, curation, standards, documentation, sharing, and archiving of data produced by grantees. Nonprofit funders are encouraged to use the goals outlined in this paper as a framework for such guidelines or policies. When possible, nonprofit funders should support the training of grantees on the tools and methodologies for sharing and appropriately analyzing data.

#### Goal #5: Promote and Support the Development and Adoption of Standards, Standard Language, and Common Data Elements

Standards relevant to research data sharing encompass a number of types, including common data models; transport or exchange formats; metadata standards and analysis standards; data elements; terminologies and vocabularies, ontologies, and code lists. Organizations such as the Clinical Data Interchange Standards Consortium, TransCelerate, and the Critical Path Institute continue to develop and maintain the data standards, tools, and methods needed to support clinical trial data sharing [31-33]. Nonprofit funders should join other stakeholders to promote the use of standards during data collection, enabling easily discoverable, searchable, interoperable, and reproducible results [10]. Disease-specific nonprofit funders can lead to defining and promoting common data elements specific to their disease of interest but should also work across disease areas to find commonalities across diseases. The development of unique data elements for a particular disease has been the norm; however, unexpected connections across diseases and treatments have been found, and ensuring a level of interoperability and comparability can only increase the power of the data being collected. Common data elements typically present a question and valid answers to be recorded in a case report form and often specify the method to be used in collecting measurements. To enable comparisons of studies that apply the same measurement method, funders can also encourage the use of standardized protocols. Funding organizations may also issue manuals on exactly how a test should be conducted (eg, the Timed 25 Foot Walk in multiple sclerosis) in the context of a clinical trial.

#### Goal #6: Include Incentives and Enforce Requirements in Grants, Contracts, and Other Funding Structures, Which Promote and Provide Accountability for Investigators to Share and Use Shared Data

Nonprofit funders should, at a minimum, require that investigators have a data sharing plan in place before enrollment of the first participant that dictates when, what, with whom, how, and under what circumstances the data will be shared. Nonprofit funders can direct grantees to applicable guidelines for specific provisions of data sharing plans, in accordance with the needs of each nonprofit funders’ organization, in general, and the clinical trial at hand, in particular [11-14]. For some communities and trials, open access to data might be appropriate, whereas others will require more limited or tiered access.

The need to improve incentives has also been emphasized by others, and nonprofit funders are in a unique position to incentivize researchers by promoting the recognition of investigators who share their data [24,25]. Funders should encourage applicants to include shared data sets as part of the biographical sketch in a proposal. Furthermore, they should instruct reviewers of proposals to view a track record of shared data sets as a positive achievement, demonstrating research productivity.

Data are valuable and often one of the greatest assets a nonprofit organization has. However, nonprofits are encouraged to maintain a clear focus on their mission and resist the temptation to unnecessarily keep data internal to their organization. Nonprofit funders should consider how to incentivize grantees to use externally authored shared data sets to improve the proposed clinical research design (eg, to better estimate the effect size of the proposed treatment; Textbox 2). This reuse of existing data sets further emphasizes the above goals of technical infrastructure, standards, common data elements, and sufficient documentation for grantees to integrate and reproduce results.

**Mechanisms for nonprofit funders to incentivize data sharing or the use of shared data sets in new research.**

**To incentivize data sharing or using shared data sets in new research, nonprofit funders could:**

1. Publish a list of top data sharers and shared data users for usage analytics, either within their organization or in partnership with other organizations.
2. Highlight the success stories of data sharing or the use of shared data by grantees in articles in high-impact publications.
3. Provide credit and/or rewarding grantees who share data and use shared data.
4. Engage in open science efforts that incentivize the use of publicly available data, crowdsource challenges in medicine, and share the data and insights gleaned from the work (eg, DREAM Challenges) [15].
5. Educate both patient and research communities on the benefits of data sharing to create an expectation of data sharing in clinical research.

#### Goal #7: Provide Funding for Data Sharing and Include This Activity as a Line Item in Grants and Contracts

Implementing data sharing involves costs, but the precise cost is often a challenge to unearth. Nonprofit funders face difficult decisions, including whether to prioritize and pay for data sharing, thereby funding fewer new grants for research. Nonprofit funders could include, as a provision in grants and contracts, the allocation of funds to support sharing the data produced by the funded research. This could entail a line item dedicating a set amount of funding, or a percentage of overall funding, to data sharing efforts. Even if they do not provide the full funding for a particular trial, organizations may use their leverage to insist on such terms in grants and contracts before promoting trials to their networks. Organizations are broadly encouraged to incorporate data sharing costs into their respective funding models, maintaining a diverse, mission-aligned portfolio. If practical, and conducive to the organization’s policies, charging a fee for access to data could help defray the cost of data sharing.

Mechanisms by which nonprofit funders could *enforce* the adoption of data sharing agreements in grants and contracts or the use of shared data sets in research design could include:

1. Withholding a portion of the allocated funding until certain benchmarks of data sharing plans are realized
2. Including a neutral third party as an honest broker to administer the sharing of data in a responsible manner
3. Promoting the clinical trial to the nonprofit funder’s patient or disease advocacy networks contingent on inclusion of language stipulating a data sharing plan
4. Requiring a data sharing plan as part of any funding request, which includes an appropriate level of detail demonstrating specific steps to comply with the funder’s data sharing requirements

In addition, nonprofit funders may facilitate the adoption of data sharing agreements by offering examples of organizations providing funding to support data sharing, such as NIH (eg, NIH grantees are permitted to charge the salaries of administrative and clerical staff as a direct cost to help investigators meet their responsibilities under the NIH policy on reporting research results) [7].

#### Goal #8: Incorporate Previous Data Sharing as a Measure of Impact When Making Decisions on Whether to Fund or Support Clinical Trials

As part of the decision-making process surrounding funding or support of clinical trials, nonprofit funders should request that prospective grantees with a history of sharing data provide evidence and impact of previous data sharing and, to the extent possible, invite grantees to provide evidence of the impact of earlier data sharing. Such evidence could include new collaborations, publications, novel analysis or findings, or the evidence that emerges from secondary monitoring of usable spaces to see who is active in the community and contributing to knowledge generation. Nonprofit funders might consider conducting pilot projects to evaluate the feasibility and identify the challenges of including prior data sharing as an impact measure.

## Discussion

The goals in this paper convey the complexity of the opportunities and challenges facing nonprofit funders and seek to provide a starting point for a data sharing *toolkit* for nonprofit funders to provide the clarity of mission and mechanisms to enforce data sharing practices their communities already expect are happening. Simply requiring data sharing by grantees would be insufficient—nonprofit funders and the communities they represent expect high-quality sharing efforts that go beyond *check-the-box* exercises. A *toolkit* for nonprofit funders might include guidance, templates, general information, and other additional resources that might be of benefit to nonprofit funders—all of which could be informed, in part, by the goals in this paper.

There are costs associated with data sharing activities; therefore, it will be important to ensure that the aspirational goals expressed in this paper do not create an undue burden on researchers, nonprofit funders, and trial participants. Some of the data sharing approaches that have been developed by the participant and advocacy community may help relieve investigators of administrative burden by streamlining data storage, curation, sharing, and archiving processes. In addition, data sharing platforms have the potential to improve aspects of clinical trial research that go beyond data sharing alone (eg, enhancing participant recruitment, engagement, retention, and encouraging collaboration).

Nonprofit funders, along with other key stakeholders, play an important role in ensuring the responsible sharing of clinical trial data. The goals in this paper offer a path forward for nonprofit funders to continue to take steps, even if incremental, toward a more robust data sharing ecosystem.

### Organizations Supporting These Goals

- Alzheimer’s Association
- Center for Open Science
- Genetic Alliance
- Critical Path Institute
- Doris Duke Charitable Foundation
- Food Allergy Research & Education
- Geoffrey Beene Foundation Alzheimer’s Initiative
- Global Healthy Living Foundation
- Michael J. Fox Foundation for Parkinson’s Research
- Multiple Myeloma Research Foundation
- National Health Council
- National Multiple Sclerosis Society
- National Psoriasis Foundation
- New York Stem Cell Foundation
- Open Humans Foundation
- Pancreatic Cancer Action Network
- Parent Project Muscular Dystrophy
- Parkinson’s Foundation
- Parkinson’s UK
- Susan G. Komen
- The V Foundation for Cancer Research
- Vivli

## Abbreviations

FAIR: findable, accessible, interoperable, and reusable
IOM: Institute of Medicine
IPD: individual participant data
NIH: National Institutes of Health
PEER: Promise for Engaging Everyone Responsibly
