# Correction: Data Sharing Goals for Nonprofit Funders of Clinical Trials

**DOI:** 10.2196/31371

**Published:** 2021-06-30

**Authors:** Timothy Coetzee, Mad Price Ball, Marc Boutin, Abby Bronson, David T Dexter, Rebecca A English, Patricia Furlong, Andrew D Goodman, Cynthia Grossman, Adrian F Hernandez, Jennifer E Hinners, Lynn Hudson, Annie Kennedy, Mary Jane Marchisotto, Lynn Matrisian, Elizabeth Myers, W Benjamin Nowell, Brian A Nosek, Todd Sherer, Carolyn Shore, Ida Sim, Luba Smolensky, Christopher Williams, Julie Wood, Sharon F Terry

**Affiliations:** 1 National Multiple Sclerosis Society Cherry Hill, NJ United States; 2 Open Humans Foundation San Diego, CA United States; 3 Novartis Basel Switzerland; 4 Edgewise Therapeutics Boulder, CO United States; 5 Parkinson’s UK London United Kingdom; 6 National Academies of Sciences, Engineering, and Medicine Washington, DC United States; 7 Parent Project Muscular Dystrophy Bethesda, MD United States; 8 Department of Neurology University of Rochester Medical Center Rochester, NY United States; 9 Biogen Cambridge, MA United States; 10 Duke University School of Medicine Durham, NC United States; 11 Bison Ink Washington, DC United States; 12 Critical Path Institute Tucson, AZ United States; 13 MJM Advisory, LLC New York, NY United States; 14 Pancreatic Cancer Action Network Washington, DC United States; 15 Doris Duke Charitable Foundation New York, NY United States; 16 Global Healthy Living Foundation Upper Nyack, NY United States; 17 Center for Open Science Charlottesville, VA United States; 18 The Michael J Fox Foundation for Parkinson’s Research New York, NY United States; 19 Department of Medicine University of California San Francisco San Francisco, CA United States; 20 Multiple Myeloma Research Foundation Norwalk, CT United States; 21 Vivli Columbia, MO United States; 22 Genetic Alliance Washington, DC United States

In “Data Sharing Goals for Nonprofit Funders of Clinical Trials” (J Particip Med 2021;13(1):e23011) the authors noted two errors.

In the originally published manuscript, Lynn Matrisian was the last author listed in the order of authorship. This has been corrected to place Lynn Matrisian fifteenth in the order of authorship, after Mary Jane Marchisotto and before Elizabeth Myers. Author affiliations have been renumbered accordingly.

As well, the affiliation for Mary Jane Marchisotto has been corrected from:

MJM Advisory, LLC, McLean, VA, United States

to:

MJM Advisory, LLC, New York, NY, United States

The complete list of authors and affiliations in the originally published paper was as follows:

Timothy Coetzee^1^, MSc, PhD; Mad Price Ball^2^, PhD; Marc Boutin^3^, JD; Abby Bronson^4^, MBA; David T Dexter^5^, PhD; Rebecca A English^6^, MPH; Patricia Furlong^7^, BSN; Andrew D Goodman^8^, MD; Cynthia Grossman^9^, PhD; Adrian F Hernandez^10^, MD, MHS; Jennifer E Hinners^11^, MPH, MD; Lynn Hudson^12^, PhD; Annie Kennedy^7^, BSc; Mary Jane Marchisotto^13^, MBA; Elizabeth Myers^14^, PhD; W Benjamin Nowell^15^, PhD; Brian A Nosek^16^, PhD; Todd Sherer^17^, PhD; Carolyn Shore^6^, PhD; Ida Sim^18^, PhD, MD; Luba Smolensky^17^, BA; Christopher Williams^19^, MSc; Julie Wood^20^, BA; Sharon F Terry^21^, MA, LhD; Lynn Matrisian^22^, PhD, MBA

^1^National Multiple Sclerosis Society, Cherry Hill, NJ, United States

^2^Open Humans Foundation, San Diego, CA, United States

^3^Novartis, Basel, Switzerland

^4^Edgewise Therapeutics, Boulder, CO, United States

^5^Parkinson’s UK, London, United Kingdom

^6^National Academies of Sciences, Engineering, and Medicine, Washington, DC, United States

^7^Parent Project Muscular Dystrophy, Bethesda, MD, United States

^8^Department of Neurology, University of Rochester Medical Center, Rochester, NY, United States

^9^Biogen, Cambridge, MA, United States

^10^Duke University School of Medicine, Durham, NC, United States

^11^Bison Ink, Washington, DC, United States

^12^Critical Path Institute, Tucson, AZ, United States

^13^MJM Advisory, LLC, McLean, VA, United States

^14^Doris Duke Charitable Foundation, New York, NY, United States

^15^Global Healthy Living Foundation, Upper Nyack, NY, United States

^16^Center for Open Science, Charlottesville, VA, United States

^17^The Michael J Fox Foundation for Parkinson’s Research, New York, NY, United States

^18^Department of Medicine, University of California San Francisco, San Francisco, CA, United States

^19^Multiple Myeloma Research Foundation, Norwalk, CT, United States

^20^Vivli, Columbia, MO, United States

^21^Genetic Alliance, Washington, DC, United States

^22^Pancreatic Cancer Action Network, Washington, DC, United States

The complete list of authors and affiliations in the corrected paper is as follows:

Timothy Coetzee^1^, MSc, PhD; Mad Price Ball^2^, PhD; Marc Boutin^3^, JD; Abby Bronson^4^, MBA; David T Dexter^5^, PhD; Rebecca A English^6^, MPH; Patricia Furlong^7^, BSN; Andrew D Goodman^8^, MD; Cynthia Grossman^9^, PhD; Adrian F Hernandez^10^, MD, MHS; Jennifer E Hinners^11^, MPH, MD; Lynn Hudson^12^, PhD; Annie Kennedy^7^, BSc; Mary Jane Marchisotto^13^, MBA; Lynn Matrisian^14^, PhD, MBA; Elizabeth Myers^15^, PhD; W Benjamin Nowell^16^, PhD; Brian A Nosek^17^, PhD; Todd Sherer^18^, PhD; Carolyn Shore^6^, PhD; Ida Sim^19^, PhD, MD; Luba Smolensky^18^, BA; Christopher Williams^20^, MSc; Julie Wood^21^, BA; Sharon F Terry^22^, MA, LhD

^1^National Multiple Sclerosis Society, Cherry Hill, NJ, United States

^2^Open Humans Foundation, San Diego, CA, United States

^3^Novartis, Basel, Switzerland

^4^Edgewise Therapeutics, Boulder, CO, United States

^5^Parkinson’s UK, London, United Kingdom

^6^National Academies of Sciences, Engineering, and Medicine, Washington, DC, United States

^7^Parent Project Muscular Dystrophy, Bethesda, MD, United States

^8^Department of Neurology, University of Rochester Medical Center, Rochester, NY, United States

^9^Biogen, Cambridge, MA, United States

^10^Duke University School of Medicine, Durham, NC, United States

^11^Bison Ink, Washington, DC, United States

^12^Critical Path Institute, Tucson, AZ, United States

^13^MJM Advisory, LLC, New York, NY, United States

^14^Pancreatic Cancer Action Network, Washington, DC, United States

^15^Doris Duke Charitable Foundation, New York, NY, United States

^16^Global Healthy Living Foundation, Upper Nyack, NY, United States

^17^Center for Open Science, Charlottesville, VA, United States

^18^The Michael J Fox Foundation for Parkinson’s Research, New York, NY, United States

^19^Department of Medicine, University of California San Francisco, San Francisco, CA, United States

^20^Multiple Myeloma Research Foundation, Norwalk, CT, United States

^21^Vivli, Columbia, MO, United States

^22^Genetic Alliance, Washington, DC, United States

The correction will appear in the online version of the paper on the JMIR Publications website on June 30, 2021, together with the publication of this correction notice. Because this was made after submission to PubMed, PubMed Central, and other full-text repositories, the corrected article has also been resubmitted to those repositories.

